# Composition of fatty acids in the maternal and umbilical cord plasma of adolescent and adult mothers: relationship with anthropometric parameters of newborn

**DOI:** 10.1186/1476-511X-11-157

**Published:** 2012-11-15

**Authors:** Olívia RC Oliveira, Michelle G Santana, Flávia S Santos, Felipe D Conceição, Fátima LC Sardinha, Glória V Veiga, Maria G Tavares do Carmo

**Affiliations:** 1 Nutritional Biochemical Laboratory, Josué de Castro Institute of Nutrition, Federal University of Rio de Janeiro, Rio de Janeiro, Brazil; 2 Department of Social and Applied Nutrition, Josué de Castro Institute of Nutrition, Federal University of Rio de Janeiro, Rio de Janeiro, Brazil; 3 Instituto de Nutrição Josué de Castro, UFRJ – Laboratório de Bioquímica Nutricional, Av. Brigadeiro Trompowski, s/n - CCS, Bloco J, 2° andar, Cidade Universitária, Ilha do Fundão, Cep.: 21941-590, Rio de Janeiro, Brazil

**Keywords:** Fatty acids, Pregnancy, Adolescence, Adulthood, Newborn

## Abstract

**Background:**

Considering the importance of long chain polyunsaturated fatty acids to fetal development and the lack of studies that have compared the status of fatty acids between adolescents and adults mothers, the purpose of this study was to evaluate the composition of fatty acids in maternal and umbilical cord plasma from adolescent and adults mothers.

**Methods:**

Forty pregnant adolescents and forty pregnant adults were selected to assess the distribution profile of fatty acids in the maternal and umbilical cord plasma. Quantification of fatty acids in the total lipids of the sample groups was performed through the use of gas-liquid chromatography.

**Results:**

The maternal and umbilical cord plasma of the adolescents showed a greater concentration of AA than did that of the adults (P < 0.05). However, a greater percentage of EPA was found in the umbilical cord plasma of the adults (P < 0.05). DHA in the plasma of the adolescent mothers correlated positively to birth weight and head circumference.

**Conclusions:**

This suggests that in situations of greater nutritional risk, as in adolescent pregnancy, n-3PUFA concentrations have a greater influence on the proper development of newborns. Moreover, variations in fatty acid concentrations in the maternal and cord plasma of adolescents and adults may indicate that pregnancy affects the LC-PUFA status of adults and adolescents in distinct ways.

## Background

The essential fatty acids (EFAs), linoleic acid (LA) and α-linolenic acid (ALA), and their long-chain derivatives (long-chain polyunsaturated fatty acids – LC-PUFA) have been identified as important determinants of fetal growth and development [1]. However, EFAs cannot be synthesized by mammalian tissue due to a lack of Δ15 and Δ12 desaturase enzymes and must be obtained through dietary intake [2]. Although human beings are incapable of performing *de novo* n-3 and n-6 fatty acid synthesis [3], they are able to desaturate and elongate them by way of other enzymes, transforming LA to archidonic acid (AA) and ALA to eicosapentaenoic acid (EPA) and docosahexaenoic acid (DHA) [4].

AA is fundamental for fetal growth [5] and DHA, in particular, plays an important role in brain and retina formation, development, and functioning during pregnancy and the early years of life. Among the n-3 series of fatty acids, DHA is found in the greatest abundance in the central nervous system of mammals and is especially concentrated in the phospholipids of the cerebral grey matter membrane and the visual components of the retina [6].

Since the publishing of a consensus in 2007, it has been recommended that pregnant and lactating women consume a daily average of 0.2 g of DHA [7], which is associated with having a beneficial effect on the development of visual acuity and cognitive function, among other neural functions in infants [8].

It is noteworthy that the n-6 and n-3 fatty acids accumulated in the fetus are essentially derived from the mother by way of placental transfer [9]. Thus, maternal dietary intake of these fatty acids should be enough to guarantee adequate availability to the fetus [10].

*Trans* fatty acids (*t*FA) are unsaturated and have at least one double bond in the *trans* configuration. They are primarily the result of the industrial hydrogenation of vegetable oils and are not synthesized by humans [11]. Like EFAs and LC-PUFAs, *trans*, too, are transferred to the fetus. However, they can impair fetal development [12].

Van Eijsden et al. [13] found a positive association between birth weight and LC-PUFA concentrations in maternal plasma during early pregnancy. The authors suggest that the dimensions of newborns may be optimized by maternal LC-PUFA intake during pregnancy.

Several studies [14-16] have identified poor dietary habits in adolescents, predominantly the high consumption of foods rich in saturated fatty acids (SFA) and *t*FA (like cookies, chocolate, and ice cream, among others). Furthermore, fish is included in the least-frequently-consumed-food group, which contributes to the findings of inadequate levels of n-3 series fatty acids [17]. Thus, adolescents are often referred to as the age group with the highest prevalence of nutritional deficiency [18].

In this context, adolescent pregnancy is associated with an increase in risk for metabolic and nutritional status problems as compared to pregnancy during adulthood [19]. Adolescent mothers are at greater risk for having children with low birth weights, which is associated with neonatal mortality [20]. Seen in this light, the proposition of this study is to compare the fatty acid profile in adolescent and adult mothers, in maternal and fetal biological compartments, and its relationship to the growth parameters of newborns in Brazil.

## Results

The average age of the pregnant adolescents was 16.7 ± 1.4 years, while that of the adults was 28.3 ± 5.7 years. The socioeconomic and demographic characteristics of the group of women studied are shown in Table 1. Analysis of the numbers related to per capita income greater than or equal to one minimum-wage salary indicated a significant difference (P < 0.05) between adolescent and adult mothers. Furthermore, more than half the adolescents evaluated (55.6%) reported having left to study due to their pregnancy.

**Table 1:** Characterization of adolescent and adult mothers regarding the socio-economic, demographic and prenatal care variables

**SOCIO-ECONOMIC VARIABLES**	**Adolescents**		**Adults**	
	**n (%)**	**CI 95%**	**n (%)**	**CI 95%**
**Per capita family income**^1^				
< 1 per capita minimum wage^2^	25 (80.6)	[62.5 – 92.5]	20 (52.6)	[35.8 – 69.0]
≥1 per capita minimum wage^2^	6 (19.4)	[7.4 – 37.5]	18 (47.4)	[40.0 – 64.2]*
**Education**				
< 8 years	4 (10)	[2.8 – 23.7]	9 (22.5)	[10.8 – 38.4]
≥ 8 years	36 (90)	[76.3 – 97.2]	31 (77.5)	[61.6- 89.2]
**Work**?				
Yes	4 (10)	[2.8 – 23.7]	25 (62.5)	[45.8 – 77.3]*
**Marital status**				
Single	32 (80)	[64.4 – 90.9]	7 (17.5)	[7.3 – 32.8]*
Married	8 (20)	[9.0 – 35.6]	33 (82.5)	[67.2 – 92.7]*
**Prenatal medical consultations**				
< 6 consultations	6 (15)	[5.7 – 29.8]	2 (5.4)	[0.7 – 18.2]
≥ 6 consultations	34 (85)	[70.2 – 94.3]	35 (94.6)	[81.8 – 99.3]

*different from adolescents (P < 0.05).

^1^Total family income / number of family members.

^2^Used the minimum wage relating to the period of data collection.

CI: confidence interval.

For the family income variable there was a loss of nine adolescents and two adults, and for the variable prenatal medical consultations there was a loss of 3 adults.

Regarding the anthropometric data of the newborns, it was found that 2.5% of the children generated by adolescent mothers had low birth weights (< 2,500 g). When birth weight was classified according to gestational age, 25% of the infants born to these women were identified as being small for gestational age (SGA). Among the adult mothers, none of them gave birth to infants with a birth weight of less than 2,500 g, however, 12.5% were classified as being SGA. Additionally, the children of both the adolescent and adult mothers mostly presented head circumference at birth within the range of adequacy (P3-P97). The same was found for length at birth (Table 2).

**Table 2:** Characterization of adolescent and adult mothers and their newborns regarding obstetric and anthropometric variables

**MATERNAL ANTHROPOMETRIC VARIABLES**	**Adolescents**		**Adults**	
	**n (%)**	**CI 95%**	**n (%)**	**CI 95%**
**Pre-gestational nutritional status**				
Underweight	3 (7.7)	[1.6 – 20.9]	1 (2.5)	[0.1 – 13.2]
Eutrophic	31 (79.5)	[63.5 – 90.7]	30 (75)	[58.8 – 87.3]
Overweight	4 (10.2)	[2.9 – 24.2]	8 (20)	[9.0 – 35.6]
Obesity	1 (2.6)	[0.1 – 13.5]	1 (2.5)	[0.1 – 13.2]
**Total**	39^a^ (100)		40 (100)	
**Adequacy of gestational weight gain**				
Insufficient	13 (33.3)	[19.1 – 50.2]	12 (31.6)	[17.5 – 48.6]
Adequate	15 (38.5)	[23.4 – 55.4]	10 (26.3)	[13.4 – 43.1]
Excessive	11 (28.2)	[15.0 – 44.9]	16 (42.1)	[26.3 – 59.2]
**Total**	39^a^ (100)		38^a^ (100)	
**DELIVERY AND NEWBORN ANTHROPOMETRIC VARIABLES**	**n (%)**	**CI 95%**	**n (%)**	**CI 95%**
**Kind of delivery**				
Cesarean	11 (27.5)	[14.6 – 43.9]	19 (47.5)	[31.5 – 63.9]
Vaginal	25 (62.5)	[45.8 – 77.3]	19 (47.5)	[31.5 – 63.9]
Forceps	4 (10)	[2.8 – 23.7]	2 (5)	[0.6 – 16.9]
**Total**	40 (100)		40 (100)	
**Birth weight** (**g**)				
< 2500	1 (2.5)	[0.1 – 13.2]	0 (0)	[0.0 – 8.8]
2500-4000	36 (90)	[76.3 – 97.2]	37 (92.5)	[79.6 – 98.4]
> 4000	3 (7.5)	[1.6 – 20.4]	3 (7.5)	[1.6 – 20.1]
**Total**	40 (100)		40 (100)	
**Head circumference at birth** (**percentile**)				
<P3	2 (5)	[0.6 – 16.9]	1 (2.6)	[0.1 – 13.8]
P3-P97	35 (87.5)	[73.2 – 95.8]	32 (84.2)	[68.8 – 94.0]
>P97	3 (7.5)	[1.6 – 20.4]	5 (13.2)	[4.4 – 28.1]
**Total**	40 (100)		38^a^ (100)	
**Length at birth** (**percentile**)				
<P3	1 (2.5)	[0.1 – 13.2]	4 (10.5)	[2.9 – 24.8]
P3-P97	37 (92.5)	[79.6 – 98.4]	34 (89.5)	[75.2 – 97.1]
>P97	2 (5)	[0.6 – 16.9]	0 (0)	[0.0 – 9.2]
**Total**	40 (100)		38^a^ (100)	
**Birth weight according to gestational age**				
SGA	10 (25)	[12.7 – 41.2]	5 (12.5)	[4.2 – 26.8]
AGA	27 (67.5)	[50.9 – 81.4]	33 (82.5)	[67.2 – 92.7]
LGA	3 (7.5)	[1.6 – 20.4]	2 (5)	[0.6 – 16.9]
**Total**	40 (100)		40 (100)	

^a^ sample number of protocols completed for the variable.

SGA: small for gestational age; AGA: adequate for gestational age; LGA: large for gestational age.

The relative concentrations of fatty acids identified in the total lipids of the maternal and umbilical cord plasma of the adolescents and adults is described in Table 3.

**Table 3:** Composition of fatty acids (%) in maternal and umbilical cord plasma of adolescent and adult Brazilian mothers

**Fatty acids**	**Maternal plasma**		**Umbilical Cord plasma**	
	**Adolescents**	**Adults**	**Adolescents**	**Adults**
**Monounsaturated**				
C18:1 (n-9) cis	17.3 ± 5.2	18.0 ± 3.8	15.7 ± 2.4	17.8 ± 7.4
C18:1 (n-9) *trans*	0.7 ± 0.6	0.6 ± 0.4	0.5 ± 0.5^a^	0.5 ± 0.5
**Essential fatty acids**				
C18:2 n-6 (linoleic acid)	31.3 ± 3.9	31.4 ± 6.0	12.5 ± 2.1^a^	12.8 ± 4.8^b^
C18:3 n-3 (α-linolenic acid)	0.2 ± 0.3	0.2 ± 0.1	0.5 ± 0.6	0.5 ± 1.3
**Long**-**chain polyunsaturated fatty acids**				
C20:4 n-6 (AA)^1^	5.5 ± 1.2	4.6 ± 2.4^a^	13.1 ± 2.4^a^	10.7 ± 4.0^b,c^
C20:5 n-3 (EPA)^2^	0.4 ± 0.2	0.4 ± 0.2	0.6 ± 0.3^a^	1.1 ± 0.8^b,c^
C22:6 n-3 (DHA)^3^	1.3 ± 0.5	1.5 ± 0.6	2.8 ± 0.7^a^	2.7 ± 1.0^b^
**MFA Total**^4^	20.3 ± 4.7	20.0 ± 3.9	21.2 ± 3.8	21.8 ± 7.2
**EFA Total**^5^	31.5 ± 3.9	31.5 ± 6.0	12.9 ± 2.3^a^	13.0 ± 4.8^b^
**n**-**6 PUFA Total**^6^	37.7 ± 4.5	35.8 ± 5.9	26.9 ± 2.4^a^	23.4 ± 5.7^b,c^
**n**-**3 PUFA Total**^7^	1.8 ± 0.7	1.9 ± 0.6	3.8 ± 1.2^a^	3.6 ± 1.9^b^
**PUFA**-**LC Total**^8^	7.2 ± 1.4	6.3 ± 2.8^a^	16.3 ± 2.7^a^	14.0 ± 4.4^b,c^
**Saturated Total**^9^	35.9 ± 3.3	39.7 ± 4.9^a^	44.5 ± 3.8^a^	46.6 ± 6.2^b^
***Trans *Total**^10^	0.8 ± 0.6	0.9 ± 0.8	0.5 ± 0.5^a^	0.8 ± 0.9

Values presented as mean and standard deviation (n = 40).

^1^AA – archidonic acid; ^2^EPA – eicosapentaenoic acid; ^3^DHA – docosahexaenoic acid; ^4^MFA total: total of monounsaturated fatty acid - It includes all the *cis* position; ^5^EFA total: total of essencial fatty acids – It includes C18:3 n-3 and C18:2 n-6; ^6^n-6 PUFA total: total of n-6 polyunsaturated fatty acids – It includes C18:2 n-6, C18:3 n-6, C20:2 n-6, C20:3 n-6, C20:4 n-6 and C22:4 n-6; ^7^n-3 PUFA total: total of n-3 polyunsaturated fatty acids – It includes C18:3 n-3, C20:5 n-3, C22:5 n-3 and C22:6 n-3; ^8^PUFA-LC total: total of long-chain polyunsaturated fatty acids – It includes EPA, DHA and AA; ^9^saturated total - It includes C14:0, C15:0, C16:0 and C18:0; ^10^*Trans* total: It includes C 18:1 n-9 *trans* and C 18:2 n-6 *trans*.

^a^ different from maternal plasma of adolescents mothers: P < 0.05.

^b^ different from maternal plasma of adults mothers: P < 0.05.

^c^different from umbilical cord plasma of adolescents mothers: P < 0.05.

Given the correlations between fatty acids in the maternal plasma of the adolescents, total SFA was shown to correlate directly to total *t*FA (r = 0.434; P < 0.01) and inversely to LA (r = -0.534; P < 0.01), total EFA (r = -0.520; P < 0.01), and to n-6 PUFA (r = -0.417; P < 0.01). A negative correlation was also found between total *t*FA and LA (r = -0.460; P < 0.01), between total *t*FA and total EFA (r = -0.419; P < 0.01) and n-6 PUFA (r = -0.369; P < 0.05), and also between relative LA and EPA levels (r = -0.450; P < 0.01).

In the umbilical cord plasma of the adolescents, total SFA showed a negative correlation with AA (r = -0.486; P < 0.01), DHA (r = -0.402; P < 0.01) and total n-6 PUFA (r = -0.360; P < 0.05). An inverse association was also noted between total *t*FA and LA (r = -0.345; P < 0.05), as well as with total EFA (r = -0.322; P < 0.05) and n-6 PUFA (r = -0.354; P < 0.05).

In the maternal plasma of the adult women, total SFA correlated positively to total *t*FA (r = 0.488; P < 0.01) and negatively to LA (r = -0.620; P < 0.01), total EFA (r = -0.625; P < 0.01) and total n-6 PUFA (r = -0.733; P < 0.01). Similarly, total *t*FA correlated negatively with the relative levels of LA (r = -0.666; P < 0.01), total EFA (r = -0.652; P < 0.01) and n-6 PUFA (r = -0.699; P < 0.01).

As for the umbilical cord plasma of the adults, total *t*FA content correlated inversely to relative AA (r = -0.466; P < 0.05) and total n-6 PUFA (r = -0.449; P < 0.05) levels. An additional inverse relationship was observed between the proportions of LA and EPA (r = -0.488; P < 0.01).

**Figure 1:**
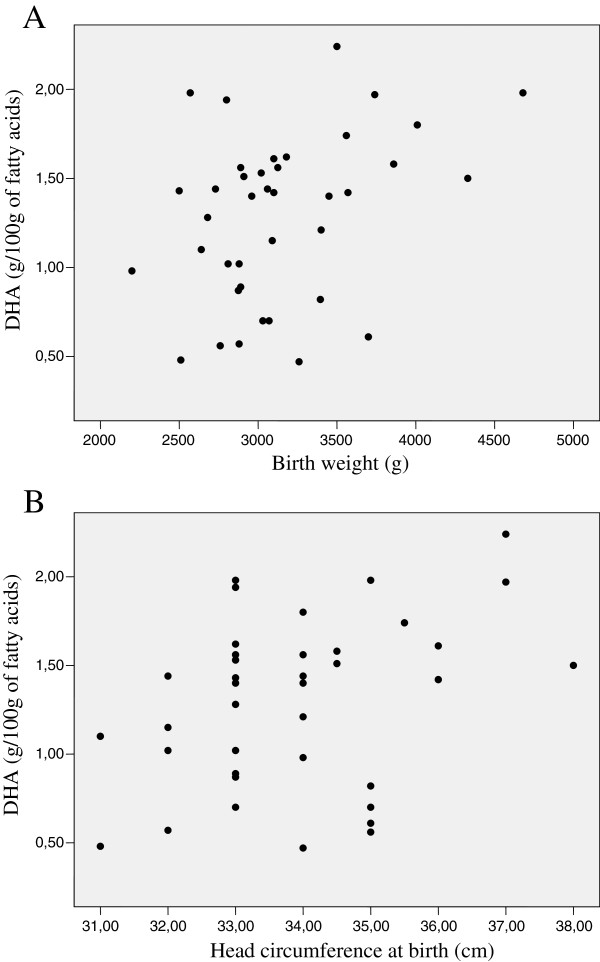
**Correlation between plasma DHA concentration of adolescent mothers and birth weight of their newborns (r = 0.374; P < 0.05) (1A).** Correlation between plasma DHA concentration of adolescent mothers and head circumference at birth of their newborns (r = 0.372; P < 0.05) (1**B**).

Among all fatty acids identified in the maternal plasma, only DHA in the adolescents correlated with the anthropometric data of their respective newborns, with a positive correlation found between this fatty acid and birth weight (r = 0.374; P < 0.05) and head circumference (r = 0.372; P < 0.05) (Figure 1).

## Discussion

According to data provided by the Brazilian Ministry of Health (MH), approximately 21% of all live births in 2007 were born of adolescents [21]. Pregnancy in this age group has been identified as a public health problem in Brazil and other countries, mainly because of possible adverse outcomes for both mother and child [22]. In this study we asked if there are differences between the fatty acid profile in maternal and fetal biological compartments of adolescent and adult mothers and if there are relationship between this profile and the growth parameters of newborns in Brazilian pregnant woman.

A large percentage of the pregnant adolescents and adults evaluated had at least six prenatal medical visits, meeting the recommendations of the MH [23]. This may have contributed to the greater proportion of newborns with adequate anthropometric nutritional status at birth in both age groups, confirming the positive association between quality of prenatal care and good outcome for the newborns as previously proposed in others studies [20,24-26].

It has been reported that the maternal and fetal fatty acid status varies widely and is influenced by the mother’s dietary lipid intake [6]. However, we found that relative concentrations of EFA in the maternal and umbilical cord plasma of both adolescents and adults were equivalent to those observed by Pankiewicz et al. [27], which were also identified for total lipids in the maternal and umbilical cord plasma of mothers.

We observed lower concentrations of LA and ALA and greater proportions of AA, EPA and DHA in the umbilical cord plasma than the maternal plasma in both age groups. These findings are consistent with the results of most previous studies which had also evaluated relative concentrations of FAs in these biological compartments [27-30]. These differences could be accounted for the presence of the placental membrane proteins which boost LC-PUFA uptake in such a way that these fatty acids are transported more efficiently than the shorter-chain variety [31]. The existence of a specific transport system for fatty acids, which includes multiple proteins, facilitates the preferential transport of LC-PUFAs via the placenta and proves to be the mechanism that allows the needs of the fetus to be met [32].

In our study the proportion of SFA was significantly higher in the umbilical cord plasma than in the maternal plasma. This is in agreement with a previous study [33]. The intense fetal lipogenesis which is reflected in the active synthesis of SFA [34] could provide the biochemical basis for these results.

Elias & Innis [29] found that dietary *t*FA intake related significantly and positively to *t*FA concentrations in the plasma of pregnant women. In addition, these authors found a significant relationship in the proportions of this fatty acid in maternal plasma and that of the umbilical cord. These findings are consistent with our results, which revealed similar concentrations of *t*FAs in the maternal and fetal plasma of the adult group. Additionally, other authors suggest that placental transfer of *t*FAs occurs selectively, because they are found in a greater percentage in the maternal plasma than the umbilical cord plasma [33,35], which is in agreement with our results for the adolescent mothers.

Both the maternal plasma and the umbilical plasma of the adolescents presented higher proportions of AA when compared to the adults. We could not find similar results in existing literature; however, one can surmise that distinct dietary intake patterns may exist between the different groups. On the other hand, considering the fundamental role that AA plays in promoting growth during the fetal and postnatal period [36], the assumption can be made that the difference found in levels of this LC-PUFA between age groups could be due to the adolescents’ stage of growth and development. Indeed, this hypothesis demands further research.

Examining the umbilical cord plasma, we found that EPA content was higher in the adults than in the adolescents. These results suggest that, comparatively, the higher AA status of the adolescents may affect mother-fetal n-3 LC-PUFA transfer, contributing to the lesser proportion of EPA in the umbilical cord plasma of this age group [37]. Previous report had pointed out that despite the scarcity of information regarding the impact of pregnancy and lactation on EFA and LC-PUFA status in adolescents, these two physiological situations may affect the status of these fatty acids differently in adults and adolescents [38].

With regard to the correlations between fatty acids in the maternal plasma of adolescents and adults, we found that total *t*FA and SFA were inversely correlated to relative concentrations of LA and to total EFA and n-6 PUFA levels. However, total *t*FA and SFA correlated positively with each other. These results can be justified by the mother’s habitual consumption of dietary sources of these fatty acids [38]. An increase in consumption of processed products and fast food, which carry elevated levels of SFAs and *t*FAs, is generally accompanied by a reduction in consumption of n-6 and n-3 FAs food sources [17]. In addition, a high intake of *t*FAs may alter maternal LC-PUFA status by reducing synthesis of AA, EPA and DHA fatty acids, especially by inhibiting the ∆6 desaturase enzyme, decreasing availability of these fatty acids to the fetus and, thus, compromising intrauterine development [39]. Proper nutritional guidance during the prenatal stage could be a strategy to overcome the potential metabolic harms of these poor dietary practices [16].

Analysis of relative FA levels in the total lipids of the adolescent maternal plasma further revealed a negative correlation between LA and EPA proportions. In this regard, it is noteworthy that the ∆5 and ∆6 desaturase enzymes that act on PUFA desaturation have a greater affinity with the more unsaturated substrates, i.e., the n-3 family, followed by the n-6 and n-9 families. However, an increase in LA availability for these enzymes may impair desaturation of ALA to EPA and to DHA [3].

The standard diet of pregnant adolescents and adults in Brazil shows a prevalence of n-6 series fatty acid food sources, like vegetable oils, mainly soy oil, as opposed to n-3 sources, like fish and fish oil, resulting in a high n-6/n-3 ratio in their diet [37,40].

Consistent with the inhibitory effect of *t*FA, as well as the fact that concentrations of this fatty acid in the fetal plasma are determined by maternal diet, a negative correlation was found between total *t*FA and total EFA and n-6 PUFA in the umbilical cord plasma of the adolescents, following the pattern of the maternal plasma. In the case of the cord plasma of the adult mothers, total *t*FA was shown to be inversely associated with AA and total n-6 PUFA, also similar to the maternal plasma of this group. Elias & Innis [29] observed an inverse relationship between dietary *t*FA and AA in the cholesterol esters of the umbilical cord plasma, as well as between *t*FA and DHA in the triacylglycerols, also in the umbilical cord plasma. It has been reported that *trans* isomers hamper placental transfer of LC-PUFA as they compete for the fatty acid-binding protein of the placental plasma membrane (FABPpm), lowering the fetal LC-PUFA status [41].

Our results do not indicate that *t*FAs interfere with the growth parameters of the newborn. These findings are consistent with data from previous studies which do not found an association between *trans* isomers and restricted intrauterine growth [12,13,29]. However, regarding n-3 LC-PUFA series, the relative concentration of DHA in the plasma of the adolescents associated positively with the weight and head circumference of the newborns. Recently, another study carried out by our group [42] found a positive and significant association between total n-3 PUFA in maternal milk and weight and length gain in preterm babies. This suggests that n-3 fatty acids have a greater influence on the proper development of the newborn, especially in situations of greater nutritional risk, like adolescent pregnancy and preterm birth.

## Conclusion

The importance of LC-PUFA’s during pregnancy is well documented [1,8,43]. The positive association between DHA and head circumference is evidence that this fatty acid is related to brain development. It was also noted that priority transfer of AA, EPA and DHA across the placenta occurs in both adolescents and adults. However, the differences in concentrations of these fatty acids in the maternal and umbilical cord plasma between adolescents and adults, indicates a need for research to identify the nature of the metabolic and physiological processes that justify these differences.

## Methods

### Participant eligibility criteria and selection

This is an observational, cross-sectional analytical study [44]. We selected 40 pregnant adolescents (between 15 and 19 years of age) and 40 pregnant adults (between 20 and 40 years of age) in two public maternities, in the city of Rio de Janeiro, Brazil. None of the subjects were smokers, pregnant with twins, drug or alcohol users, or chronic disease sufferers, nor were there any fetal complications. The protocol used to obtain socio-economic and obstetric data was developed and tested beforehand.

The project was approved by the Research Ethics Committees of two public maternity wards in the city of Rio de Janeiro, Brazil, in accordance with National Health Council resolution 196/96 [45].

### Assessment of maternal nutritional status

Pre-gestational weight (PGW) and height measurements were taken on the first visit to antenatal care, prior to the 14^th^ gestational week [46]. The final weight of pregnancy (before delivery) was measured on admission for delivery.

To evaluate the pre-gestational nutritional status of the pregnant adolescents, Body Mass Index (BMI) cut-off points were used that were specific for age and female gender [47].

The pre-gestational nutritional status of the pregnant adults was assessed on the basis of BMI cut-off points recommended by the Institute of Medicine (IOM), 2009 [48].

The adequacy of gestational weight gain was assessed according to IOM-recommended weight gain ranges (2009) [48] within the following pre-gestational BMI categories: underweight, 12.5-18.0 kg; eutrophic, 11.5-16.0 kg; overweight, 7.0-11.5 kg; and obesity, 5.0-9.0 kg (adults) and 7.0-9.1 kg (adolescents, as proposed by GUTIERREZ & KING) [49].

### Assessment of nutritional status of newborn

The classification of low birth weight (BW) was defined according to the World Health Organization established cut-off point [50] of weight less than 2,500 g. To classify BW according to gestational age, we used the Alexander graph [51]. For length and head circumference at birth, the curves published by the World Health Organization in 2006 [52] and 2007 [53], respectively, were used as references.

### Collection and storage of biological material

A 5-mL sample of maternal blood was taken intravenously by a qualified professional. Blood from the umbilical cord was collected by hand-milking before expulsion of the placenta. Both samples were collected using tubes containing 1 g Na2-EDTA/L. The material underwent centrifugation (3,500 rpm for 15 minutes) to separate and extract the plasma, which was transferred to eppendorf tubes that were then stored at a temperature of –70°C until the time of analysis.

### Quantification of fatty acids in total plasma lipids

The lipids in the samples were extracted, saponified and methylated according to the method described by Lepage & Roy [54]. Fatty acid esters were quantified through gas-liquid chromatography using the Perkin Elmer autosystem XL chromatograph, equipped with a flame ionization detector and Turbochrom software. Fatty acids were separated using an SP 2560 capillary column (Supelco, USA), with 100 m × 0.25 mm × 0.20 μm measurements. The chromatographic conditions were similar to those described by Tinoco et al. [55]. The esters were identified by comparing their retention times to known standards (Sigma, Supelco). Results were expressed as mean ± standard deviation of weight percentage (g/100 g of total fatty acids).

### Statistical analysis

The Skewness test was used to evaluate the symmetry of continuous variables. A confidence interval of 95% was used to compare frequencies between categorical variables. The paired t test and Wilcoxon test were used to compare data with parametric and nonparametric distributions, respectively, in the same group. When comparing data with symmetrical and nonsymmetrical distributions between the groups, the independent t test and Mann-Whitney test were used, respectively. To assess correlations between continuous variables, we used the Pearson or Spearman correlation coefficients, depending on the nature of the variables (normal distribution or not, respectively). To perform statistical analysis, we used the Statistical Packages for Social Sciences (SPSS) version 13.0 and Epi-Info version 6.0. Values of P < 0.05 were considered to be statistically significant.

## Abbreviations

EFAs: Essential fatty acids; LA: Linoleic acid; ALA: α-linolenic acid; LC-PUFA: Long-chain polyunsaturated fatty acids; AA: Archidonic acid; EPA: Eicosapentaenoic acid; DHA: Docosahexaenoic acid; *t*FA: *Trans* fatty acids; SFA: Saturated fatty acids; PGW: Pre-Gestational Weight; BMI: Body Mass Index; IOM: Institute of Medicine; BW: Birth weight; SGA: Small for gestational age; *n*-6 PUFA: *n*-6 polyunsaturated fatty acids; MH: Brazilian Ministry of Health; *n*-3 LC-PUFA: *n*-3 long-chain polyunsaturated fatty acids.

## Competing interests

The authors declare that they have no competing interests.

## Authors’ contributions

ORCO participated in the design of the study, acquisition of data, helped to perform the statistical analysis and drafted the manuscript. MGS participated in the acquisition of data and helped to perform the statistical analysis. FSS participated in the design of the study and in the acquisition of data. FDC carried out the quantification of fatty acids in total plasma lipids. FLCS participated in the design of the study and helped to draft the manuscript. GVV participated in the design of the study and helped to perform the statistical analysis. MGTC conceived of the study, participated in its design and coordination and helped to draft the manuscript. All authors read and approved the final manuscript.
